# New Diagnostic and Treatment Modalities for Neurogenic Thoracic Outlet Syndrome

**DOI:** 10.3390/diagnostics7020028

**Published:** 2017-05-27

**Authors:** M. Libby Weaver, Ying Wei Lum

**Affiliations:** 1 Department of Surgery, Johns Hopkins Hospital, Baltimore, MD 21287, USA; weave25@jhmi.edu; 2 Department of Surgery, Johns Hopkins Heart and Vascular Institute, Johns Hopkins Medical Centers, Baltimore, MD 21287, USA

**Keywords:** neurogenic thoracic outlet syndrome, brachial plexus compression, brachial plexopathy

## Abstract

Neurogenic thoracic outlet syndrome is a widely recognized, yet controversial, syndrome. The lack of specific objective diagnostic modalities makes diagnosis difficult. This is compounded by a lack of agreed upon definitive criteria to confirm diagnosis. Recent efforts have been made to more clearly define a set of diagnostic criteria that will bring consistency to the diagnosis of neurogenic thoracic syndrome. Additionally, advancements have been made in the quality and techniques of various imaging modalities that may aid in providing more accurate diagnoses. Surgical decompression remains the mainstay of operative treatment; and minimally invasive techniques are currently in development to further minimize the risks of this procedure. Medical management continues to be refined to provide non-operative treatment modalities for certain patients, as well. The aim of the present work is to review these updates in the diagnosis and treatment of neurogenic thoracic outlet syndrome.

## 1. Introduction

Neurogenic Thoracic Outlet Syndrome (nTOS) is a clinical diagnosis that describes the symptomatic manifestation of the compression of the neurologic structures traversing the thoracic outlet, namely the brachial plexus. It is the most common of the three subtypes of TOS representing 95% of overall TOS occurrences, with women aged 20–40 being the primary population affected, yet it is the most controversial for several reasons [1,2]. With nTOS, unlike arterial and venous TOS, diagnosis is largely clinical and subjective in nature, with no definitive imaging or diagnostic studies available to confirm its presence. Until recently, there were no clear guidelines to define specifically which patients clearly demonstrate a clinical diagnosis of nTOS. New developments in objective diagnostic studies, as well as more clearly-defined guidelines for proper diagnosis of nTOS help to better identify patients suffering from nTOS so that these patients may receive appropriate treatment. Therapeutic modalities for nTOS range from medical to operative in nature. Several surgical approaches, including supra-clavicular and trans-axillary, are utilized with equivalent rates of success. However, new surgical approaches, including video-assisted thoracoscopic, endoscopic-assisted and robotic approaches also demonstrate excellent outcomes.

## 2. Diagnostic Criteria

Until recently, a vital challenge in the efficient and adequate treatment of patients suffering from nTOS was the lack of agreed upon diagnostic criteria. The Consortium for Outcomes Research and Education of Thoracic Outlet Syndrome recently developed a preliminary set of diagnostic criteria for nTOS. This group consists of physicians and scientists from multiple disciplines working together with the intention of assisting practitioners in accurately identifying, and thus employing appropriate management strategies for, those patients presenting with symptoms suggestive of nTOS. These guidelines clearly delineate combinations of history and physical examination findings that are required to properly diagnose, and thus treat, nTOS, as outlined in Table 1. The findings must be present for a minimum of three months and must not be attributable to any other neurologic cause [3].

Additionally, the Society for Vascular Surgery published reporting standards for TOS, the primary aim of which is to provide a clear and consistent understanding and definition of what constitutes a diagnosis of nTOS, while also accurately assessing the results of various management strategies. This more simplistic definition consists of the following four criteria: signs and symptoms of pathology occurring at the thoracic outlet (pain and/or tenderness), signs and symptoms of nerve compression (distal neurologic changes, often worse with arms overhead or dangling), absence of other pathology potentially explaining the symptoms and a positive response to a properly-performed scalene muscle test injection [4]. The subjective nature of many of these diagnostic findings contributes to the controversy surrounding the validity of the diagnosis of nTOS.

## 3. Diagnostic Techniques

### 3.1. Imaging

There are objective radiologic diagnostic findings in some, but not all, patients who fit the criteria for diagnosis of nTOS. Although imaging modalities, particularly ultrasonography, are generally able to provide conclusive evidence of the presence of vascular forms of TOS, the efficacy of diagnostic imaging modalities in the evaluation of nTOS is less clear. Nonetheless, imaging may prove to be a useful adjunct in the diagnosis of nTOS. Ultrasonography, for example, may demonstrate associated vascular compression in those patients presenting with symptoms suggestive of nTOS. In one study of 143 patients with nTOS symptoms, duplex scanning demonstrated ipsilateral compression of vessels in 31% of these patients, compared to only 8% demonstrating asymptomatic contralateral compression and 10% bilateral compression when patients’ arms were placed in abducted positioning [5]. These results suggest that findings of vascular compression may be present even in nTOS, providing additional support of the diagnosis. Ultrasonography may also demonstrate signs specific to nTOS. One study describes the “wedge-sickle sign”, identification of a fibromuscular structure causing indentation of the lower trunk of the brachial plexus. The structure itself is hyper-echoic in nature, while the lower trunk is hypo-echoic due to loss of the nerve fascicle. In this study, the presence of the wedge-sickle sign was highly sensitive (95%) with a positive predictive value of 82.6% [6].

Computed Tomography (CT) and Magnetic Resonance Imaging (MRI) may be utilized to assess for compression of the brachial plexus as it traverses the thoracic outlet. They can identify bony abnormalities or fibromuscular abnormalities and anatomic variants which may predispose patients to the development of nTOS. Specifically, these imaging studies can identify abnormal branching patterns or an abnormal course of the brachial plexus, each of which may be associated with nerve compression. Dynamic changes causing narrowing of those spaces through which the brachial plexus traverses may also predispose patients to nTOS and may be identifiable with proper positioning of the patient when obtaining images [3]. The utility of MRI appears to be dependent on the specific technique utilized. One study examined 42 cases of TOS, which were managed with surgical decompression. This study demonstrated poor correlation between MRI and intraoperative findings. The sensitivity and specificity of MRI for diagnosis of TOS in this study was 41% and 33%, respectively [7]. Alternatively, MR Neurography (MRN) shows potential as a beneficial diagnostic tool for nTOS [8]. Specifically, variations of MRN such as Short Tau Inversion Recovery (STIR) sequences and the Spectral Adiabatic Inversion Recovery (SPAIR) preparatory module deliver a more complete anatomical description of the nerves comprising the brachial plexus. Additionally, the use of diffusion tensor imaging sequences to visualize nerve fascicles is employed in the modeling technique of tractography, which allows for a more comprehensive assessment of peripheral nerve injury [9]. One study using MRN demonstrated a 100% positive predictive value in all thirty patients. In this study, however, compression was also identifiable using ultrasonography in all patients with MRN-identified nerve lesions [10].

Electrodiagnostic testing is also of utility in the diagnosis of nTOS. These tests can serve to rule out other neurologic etiologies as contributors to a patient’s symptomatology. In addition, axonal loss of brachial plexus neurons is present on electrodiagnostic testing in those patients ultimately diagnosed with nTOS. When comparing the Median Antebrachial Cutaneous Nerve (MABC), which arises from T1 nerve fibers, to sensory nerve fibers derived from the level of C8, nerve conduction of the MABC demonstrates abnormal amplitudes. Thus, combined evaluation of nerve fibers originating at both levels is recommended [11]. When evaluating nTOS, several studies over the last decade suggest that the most sensitive diagnostic nerve conduction study is the demonstration of a diminished amplitude in MABC Sensory Nerve Action Potential (SNAP). One study in particular revealed abnormal MABC SNAP in 85.7% of patients diagnosed with nTOS compared to ulnar SNAP (77.8%) and median and ulnar compound muscle action potentials (55.6% and 33.3%, respectively) [12]. Still, reduced SNAP of the ulnar nerve or decreased thenar M-wave voltage are associated with impingement of the brachial plexus [3]. 

### 3.2. Scalene Injection

Another diagnostic modality that is important in the evaluation of nTOS is scalene injection. Although this technique is not new, it continues to undergo modifications that further enhance its diagnostic efficacy. Scalene injection can be a qualitative diagnostic tool that is additionally predictive of surgical outcomes in those patients under consideration for surgical management. It may also be considered as an alternative treatment modality for appropriately selected patients. In one study, work performance, power and time to fatigue were measured on patients undergoing anterior scalene muscle block with 1% lidocaine injection during a variety of exercises following the procedure. The results demonstrated statistically-significant increases in function motor capacity. This suggests that anterior scalene muscle blocks may provide quantifiable information that may assist in successful and accurate diagnosis of nTOS [13]. High-performance athletes are a special population that may require a more intense post-procedural exercise regimen to accurately assess the effect on patient symptomatology and verify a successful scalene block [14].

Further refining the diagnostic techniques outlined above, as well as developing new objective diagnostic tools, is important not only to improve accuracy and consistency in the diagnosis of nTOS, but also to allow for the diagnosis to be made in a more efficient and timely manner. In nTOS in particular, early surgical intervention following symptom onset is associated with improved patient outcomes, particularly in patients greater than forty years of age [15,16]. The utility of clinical presentation in the diagnosis of TOS, however, remains extremely important, and its value cannot be overemphasized. A retrospective review of 621 patients at one institution who were either self-referred of referred by another physician to vascular surgeons for suspicion of TOS demonstrated high diagnostic accuracy by both referring physicians and patients themselves, with 91% and 97% respectively ultimately being diagnosed with TOS [17]. This underscores the significance of recognizing clinical characteristics consistent with TOS to establish the proper diagnosis.

### 3.3. Genetics

Finally, it is worthwhile to mention the role of genetics in the diagnosis of TOS. Although no specific genetic mutations have been identified in association with the development of TOS, there is at least one case report of TOS presenting in multiple family members, suggesting the potential for a genetic predisposition to development of the syndrome [18]. In particular, variations in HOX gene expression are implicated in the development of anomalies of the axial skeleton, including the presence of a cervical first rib [19]. With the increasing use of genetics in medicine, it is possible that genetic analysis will become an important factor in the diagnosis of TOS in the future.

## 4. Treatment

### 4.1. Surgical Management

#### 4.1.1. Patient Selection and Surgical Outcomes

With proper patient selection, the operative management of nTOS has excellent outcomes. Appropriate patient selection and management is a key determining factor in surgical success. Successful stratification of patients into appropriate management protocols is accomplished with implementation of several selection strategies. Exclusion of cervical or other peripheral nerve compression syndromes is a critical component of a thorough preoperative evaluation. Patients who are less than 40 years of age, present with a shorter symptoms duration and are non-smokers have better outcomes than other patients undergoing surgical management of TOS [20,21]. One vascular surgery referral center determined only 1/3 of the 621 patients referred for surgical intervention were appropriate candidates for First Rib Resection with Scalenectomy (FRRS). This institution demonstrated a 91% surgical success rate in those who were offered operative management [20]. This institute selects patients with nTOS who are refractory to an eight-week course of physical therapy and responsive to anterior scalene muscle blocks with Botox or lidocaine for surgical intervention [1]. In contrast, another major referral center for TOS implements an approach in which nTOS patients are deemed appropriate for operative management only if they demonstrate symptomatic improvement with 8–16 weeks of physical therapy. One study from this institution reports 24 of 59 patients referred for further evaluation of nTOS were candidates for surgical intervention with a comparable rate of symptomatic improvement in 90% at one year [22]. There is evidence, however, that a subset of patients presenting with nTOS with co-existing arterial involvement is refractory to, and sometimes worsened with, physical therapy. Ultimately, these patients demonstrate even better outcomes than those with nTOS only after surgical intervention, with 100% showing improvement or resolution of neurogenic symptoms post-operatively in one study [23]. 

Anterior scalene blocks with lidocaine may be used to predict patients who will respond positively to operative intervention, particularly in patients over the age of forty. In this patient population, those who had a successful response to scalene blocks demonstrated an 81% success rate after surgery as compared to only a 67% surgical success rate in those patients who failed to respond to a scalene block pre-operatively. Response to scalene block was not as predictive of surgical success in patients under the age of 40 in this study. Additionally, patients over the age of 40 who presented with a longer duration of symptoms had a significantly lower rate of positive surgical outcomes. Patients less than forty years of age did not demonstrate this association [16]. These findings reiterate the importance of appropriate patient selection when evaluating those patients over the age of 40 for surgical management.

Assessment of the vascular structures of the thoracic outlet may also be an important component of the pre-operative evaluation of patients presenting with nTOS. Even without vascular symptoms, internal jugular and subclavian vein stenoses have a high incidence in patients presenting with nTOS. One study revealed stenosis of >66% within these vessels in up to 68% of these patients [24]. Although it is unclear what relationship this finding may have with surgical outcomes, recognition of asymptomatic vascular changes in patients presenting with neurogenic TOS symptoms may be useful information when determining patient appropriateness for surgical intervention.

#### 4.1.2. Updates in Surgical Techniques

Traditionally, surgical management of nTOS consists of scalenectomy alone versus scalenectomy in combination with resection of the first rib and/or cervical rib when applicable. Various approaches including supraclavicular, infraclavicular and transaxillary approaches are all employed with equivalent excellent outcomes achieved at high volume centers. The authors’ institution primarily utilizes the transaxillary approach for nTOS and reports a greater than 90% rate of improvement, or full resolution, of symptoms in 308 patients undergoing first rib resection and scalenectomy [20].

Although the transaxillary approach requires only a single small incision that is discretely placed in the axilla, other “minimally-invasive” approaches have been developed in recent years. Some institutions describe the use of Video-Assisted Thoracoscopic Surgery (VATS) as a minimally-invasive approach to first rib resection, with one reported advantage of this approach being a clearer visualization of the operative field, potentially minimizing injury to the neurovascular bundle. One institute utilizes a three-incision method in which two working and one scope port are placed. Although their data include a very small group of 10 patients, they did observe complete resolution of symptoms in 90% of patients, which is comparable to the success rate of other techniques. The median operative time was 85 min, and the median post-operative length of stay was 72 h [25]. A larger study examined 58 patients undergoing 66 rib resections (eight bilateral) with a different VATS technique requiring a transaxillary incision with a single port placement just below the incision. With this technique, 88.7% had resolution of headaches, although outcomes associated with other neurologic symptoms are unclear. Post-operative complications developed in 12% of the patients. These complications included surgical site infection, pneumothorax, pulmonary embolism and pneumonia. The average length of hospital stay post-operatively was 2.5 days [26].

Another minimally-invasive technique described in the literature is that of robotic first rib resection. One institution reports excellent results in five patients who underwent robotic first rib resection for venous TOS with no reported morbidities. This technique requires four incisions in total. At one-year follow-up, all patients maintained patent subclavian veins without any additional intervention. The average length of hospital stay was three days [27]. A later series from this institution evaluated the outcomes of robotic first rib resection for venous TOS in 13 patients and continued to demonstrate similar results with 100% vein patency at six months and a mean post-operative hospital stay of three days. The mean operative time in this study was 163 ± 39 min, which is longer than the average operative time of the other previously-established surgical approaches [28]. Given that most experienced centers routinely performing first rib resection with traditional approaches via the supraclavicular or transaxillary incision have a much shorter length of hospital stay of one day post-operatively and that these approaches require only a single incision, implementation of the above techniques has not yet occurred on a large scale [1].

A final novel technique that is worth mentioning is that of the endoscopic-assisted transaxillary approach. This approach aims to decrease the risk of pneumothoraces, a complication that is observed at a rate of 10–23% of patients undergoing transaxillary first rib resection [1,29]. One series of 22 patients undergoing first rib resection with the endoscopic-assisted transaxillary approach for better visualization of the operative field reported no complications associated with vascular, neural or pleural damage with success rates comparable to those of the traditional transaxillary approach [30].

Surgical complications associated with decompression of the thoracic outlet include pneumothorax, wound infection, hematoma and hemothorax. At our institution, there were no arterial, venous or nerve root injuries in ten years of treating 538 patients undergoing 594 FRRS procedures, 308 of which were for the neurogenic form of TOS specifically [20]. It should also be noted that there is evidence that surgical outcomes in those patients presenting with work-associated injuries and with workers’ compensation are worse. One study demonstrates 60% of patients remained disabled and unable to continue work-required activities at one year after surgical intervention [31].

### 4.2. Medical Management

Despite the high rate of success with minimal complications associated with surgical decompression, medical management may be the most appropriate option for certain patients. These measures are effective in up to 70% of patients presenting with nTOS. Physical therapy, modifications to daily activities to keep symptom exacerbation at a minimum and complementation of the treatment regimen with pharmacologic agents are all medical measures that may be employed in the treatment of nTOS [32]. Up to 1/3 of athletes presenting with nTOS return to full function with physical therapy alone. Duration to symptom onset may be associated with increased success of medical management, as patients in this study experienced a short duration of symptoms with a mean of three months from symptom onset to evaluation and intervention [33].

Anterior scalene muscle injection not only serves as a both diagnostic and prognostic tool; it also plays a role as a therapeutic tool in patients with nTOS. A recent study shows 88.2% of 142 patients treated with scalene injections of Marcaine and triamcinolone demonstrated symptomatic improvement or resolution. Shorter symptom duration prior to the first injection was associated with increased improvement in those patients with a traumatic etiology, while the response of patients presenting with other etiologies of TOS was not affected by symptom duration [34]. Alternatively, a double-blind, randomized, controlled trial of 38 subjects did not demonstrate significant improvement in pain in patients undergoing anterior scalene injection with Botox vs. placebo. Notably, patients enrolled in this study had a mean symptom duration of six years [35].

## 5. Summary

In conclusion, neurogenic thoracic outlet syndrome remains a challenging entity to diagnose, but demonstrates excellent outcomes once a diagnosis is confirmed and treatment initiated. Recent statements clarify the defining factors of neurogenic thoracic outlet syndrome by clearly outlining a set of criteria consistent with the diagnosis of nTOS. The development of a clear set of criteria for diagnosis will allow for further advancements in the diagnosis and management of nTOS. Imaging studies continue to evolve as new modalities with higher quality allow for the possibility of the development of objective measures for the diagnosis of nTOS. First rib resection with anterior scalenectomy remains the operation of choice for decompression, but surgical advancements continue with the use of minimally-invasive approaches. Refinement of medical management strategies continues to offer additional non-operative treatment modalities to those patients who do not prove to be good candidates for surgical intervention or who prefer not to undergo surgical intervention.

## Figures and Tables

**Table 1:** Preliminary criteria for the clinical diagnosis of Neurogenic Thoracic Outlet Syndrome (nTOS).

Unilateral or Bilateral Upper Extremity Symptoms	
(1) Extend beyond the distribution of a single cervical nerve root or peripheral nerve	
(2) Have been present for at least 12 weeks	
(3) Have not been satisfactorily explained by another condition	
(4) Meet at least one criterion in at least four of the following five categories:	
1. Principal Symptoms	1A. Pain in the neck, upper back, shoulder, arm and/or hand
	1B. Numbness, paresthesias and/or weakness in the arm, hand or digits
2. Symptom Characteristics	2A. Pain/paresthesias/weakness exacerbated with elevated arm positions
	2B. Pain/paresthesias/weakness exacerbated with prolonged or repetitive arm/hand use or by prolonged work on a keyboard or other repetitive strain
	2C. Pain/paresthesias radiate down the arm from the supraclavicular or infraclavicular space
3. Clinical History	3A. Symptoms began after occupational, recreational or accidental injury of the head, neck or upper extremity, including repetitive upper extremity strain or overuse activity
	3B. Previous clavicle or first rib fracture or known cervical rib(s)
	3C. Previous cervical spine or peripheral nerve surgery without sustained improvement
	3D. Previous conservative or surgical treatment for TOS
4. Physical Examination	4A. Local tenderness on palpation over scalene triangle or subcoracoid space
	4B. Arm/hand/digit paresthesias on palpation over scalene triangle or subcoracoid space
	4C. Weak handgrip, intrinsic muscles, or Digit 5, or thenar/hypothenar atrophy
5. Provocative Maneuvers	5A. Positive Upper Limb Tension Test (ULTT)
	5B. Positive 1- or 3-min Elevated Arm Stress Test (EAST)
